# Revisiting the Intratemporal Course of the Facial Nerve

**DOI:** 10.7759/cureus.73280

**Published:** 2024-11-08

**Authors:** Charuvi Guttal, K Prasad, Venkateshu KV, Induvarsha G, Gautham S

**Affiliations:** 1 Otolaryngology, Sri Devaraj Urs Academy of Higher Education and Research, Kolar, IND; 2 Anatomy, Sri Devaraj Urs Academy of Higher Education and Research, Kolar, IND

**Keywords:** angulation, facial nerve, facial nerve course variation, intratemporal course, mastoid segment, mastoid surgeries, stylomastoid foramen

## Abstract

Introduction

The facial nerve displays a lot of variations and anomalies in its course. Having sound knowledge about the surgical anatomy along with its intricacies is essential in mastoid surgeries. In this study, we have documented the angle of deviation of the mastoid segment of the facial nerve during its intratemporal course and the importance of this angle during mastoid surgeries.

Aim and objectives

The objectives of this study were to document and assess the orientation of the intratemporal course of the mastoid segment of the facial nerve and to measure the angle of deviation of the mastoid segment of the facial nerve as it traverses from the second genu to the stylomastoid foramen.

Methodology

A prospective observational study was conducted between December 2023 and June 2024 on 20 wet temporal bone specimens. Following canal wall down mastoidectomy, the facial nerve was explored and completely traced from the second genu to the stylomastoid foramen. High-quality images were captured and uploaded to the GIMP 2.10.36 software. At the second genu, a tangential line was drawn in the sagittal plane. Another line was drawn along the course of the facial nerve until its exit at the stylomastoid foramen. The angle between these two lines was estimated, and the angle of deviation was documented using this software and statistically analyzed.

Results

The mean angle of deviation at the second genu was found to be 15 (12-18) degrees in all 20 specimens. It was found that as the facial nerve traverses towards the stylomastoid foramen, from its anteromedial course it becomes more lateral and superficial. The anteroposterior angulation between males and females had a p-value of 0.02591, showing that it was significant. Similarly, the medial to lateral angulation between males and females had a p-value of 0.0458, which showed significance. However, the anteroposterior and medial to lateral angles did not show any significance when compared to the standard angle of 15 degrees as per literature, showing a p-value of 0.456 and 0.275 respectively. This helped us understand and document the highly variable course of the mastoid segment of the facial nerve, which can be emphasized during further research.

Conclusion

This angle of deviation of the mastoid segment of the facial nerve, around 15 degrees, will help surgeons be more careful during mastoid surgeries as it helps in assessing the depth and highly variable course of the facial nerve during its intratemporal course. This further acts as a guiding tool for budding surgeons in preventing inadvertent facial nerve injuries.

## Introduction

The facial nerve displays a lot of variations and anomalies in its course as it traverses the temporal bone from the internal acoustic meatus to the stylomastoid foramen [1]. It is divided into three parts: labyrinthine, tympanic, and mastoid segments, among which the mastoid segment of the facial nerve is highly prone to injury during its exploration [2]. The mastoid segment (13-15 mm) extends from the second genu to the stylomastoid foramen and assumes a vertical position as it drops downward in the posterior wall of the tympanic cavity and the anterior wall of the mastoid to exit at the base of the skull via the stylomastoid foramen [2]. Anatomically, this mastoid segment of the facial nerve takes a course which is posteromedial to anterolateral as it travels from the second genu to the stylomastoid foramen. The deviations that the nerve undergoes are in different coronal planes and the angles (anteroposterior and medial to lateral) that it forms during its course have not been studied to date.

Variations in its course can alter surgeries such as posterior tympanotomy and canaloplasty, which means that drilling in this area requires caution and is also prone to a high risk of facial nerve injury [3]. Thus, having sound knowledge about the surgical anatomy along with its intricacies is essential in middle ear surgery. This knowledge helps avoid complications in extensive diseases involving the mastoid and facial nerve. Kalaiarasi R et al. stated in 'Anatomical Features of Intratemporal Course of Facial Nerve and its Variations' that three-dimensional anatomical knowledge provides the foundation for safe and skillful dissection of the very complex temporal bone and tortuous facial nerve. The mastoid segment of the nerve runs in a line from the fossa incudis, where the short process of the incus lies, to the anterior end of the digastric ridge; hence, knowing these surgical landmarks becomes extremely important in surgeries like facial nerve decompression and parotid surgeries [4].

This study is conducted to assess the microscopic anatomy of the facial nerve as it courses through the intratemporal region and its angle of deviation as it traverses from the second genu to the stylomastoid foramen. This is crucial for surgeons to avoid facial nerve injury.

## Materials and methods

**Figure 1:**
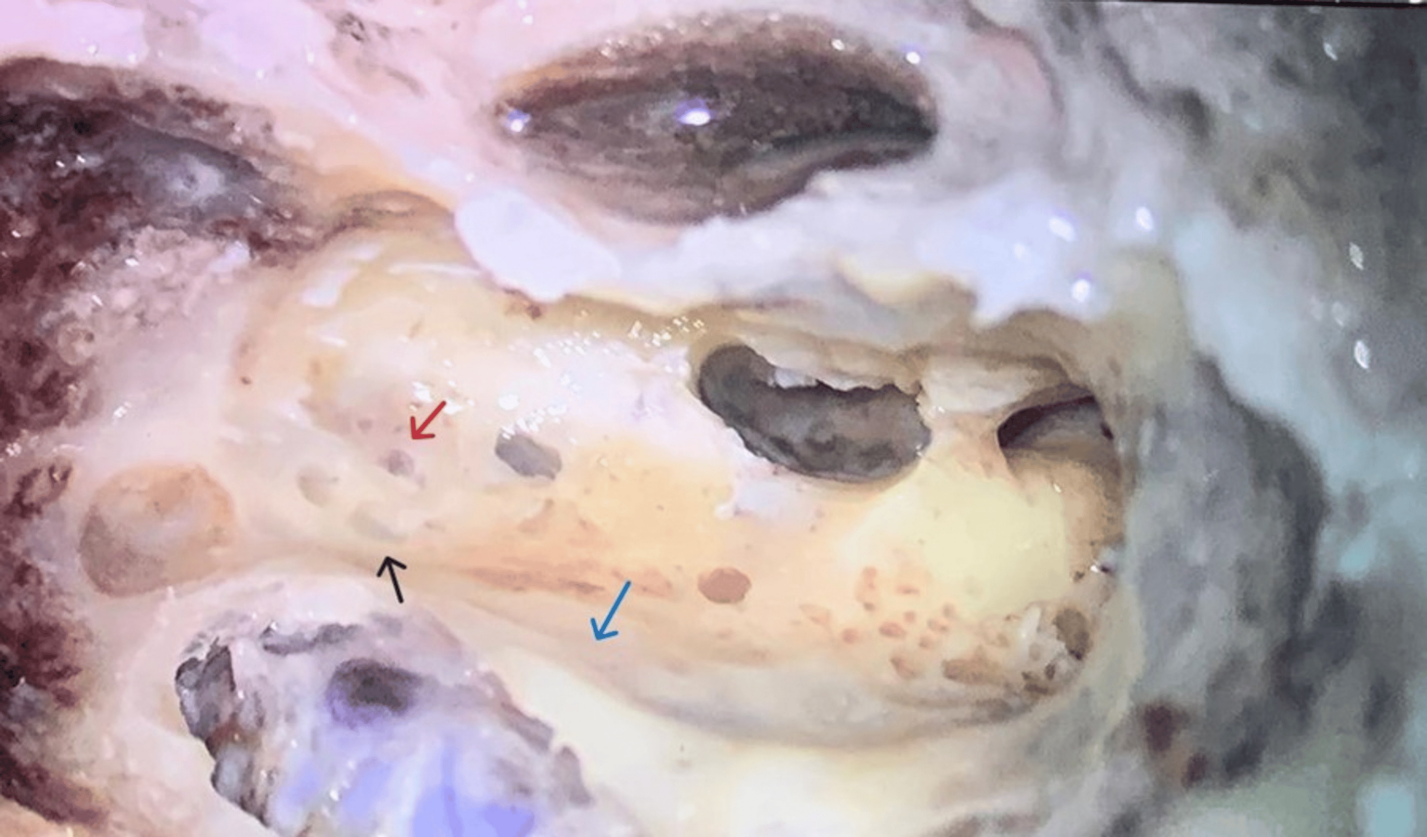
Left temporal bone showing cortical mastoidectomy. (a) Black arrow: non-ampullary end of superior semicircular canal. (b) Red arrow: ampullary end of lateral semicircular canal. (c) Blue arrow: sigmoid sinus.

This was a prospective observational study approved by the Institutional Ethics Committee of Sri Devaraj Urs Medical College, Kolar, India, with IEC no. SDUMC/KLR/IEC/538/2023-24 on January 19, 2024. This study was conducted from December 2023 to June 2024, during which 20 wet temporal bone cadavers preserved in 10% formalin were dissected under a microscope. In these specimens, cortical mastoidectomy was performed, after which the dural plate was delineated superiorly, the sinus plate posteriorly, and the sinodural angle was exposed. The lateral semicircular canal was delineated in the floor and the digastric ridge inferiorly (Figure 1).

**Figure 2:**
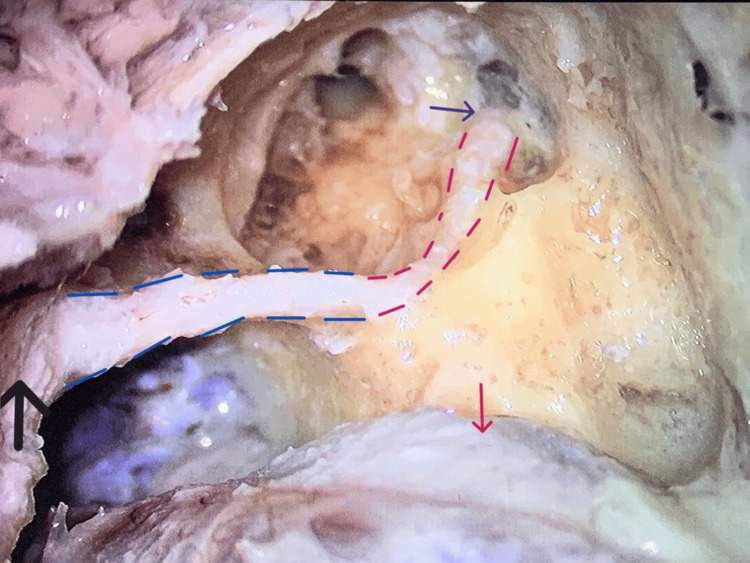
Facial canal traced in its complete length: (a) Tympanic segment (pink dotted line) and Mastoid segment (blue dotted line). (b) Purple arrow: second genu. (c) Black arrow: opening of stylomastoid foramen. (d) Red arrow: sigmoid sinus bulge.

The posterior canal wall was then reduced, the anterior and posterior buttress removed, and the facial ridge lowered [5]. The facial bony canal was then drilled to expose the facial nerve, which was completely traced from the second genu to the stylomastoid foramen (Figure 2).

**Figure 3:**
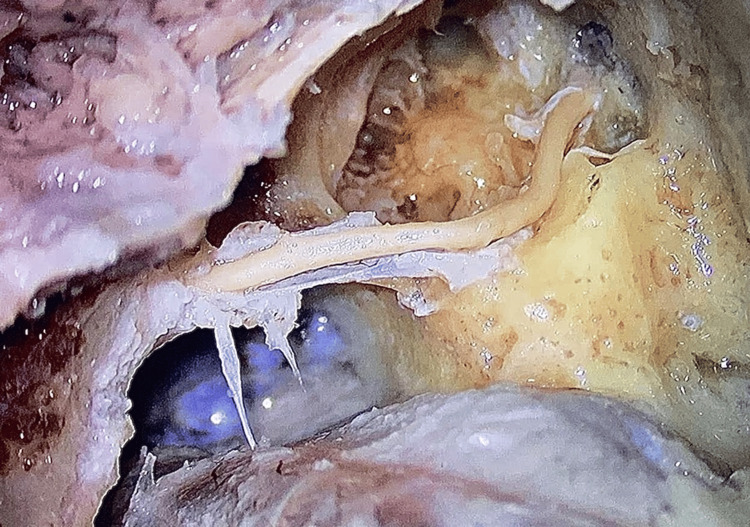
Exposure of the facial nerve after removing the bony canal and nerve sheath.

​​​The facial nerve sheath was removed, and the facial nerve was completely traced (Figure 3).

**Figure 4:**
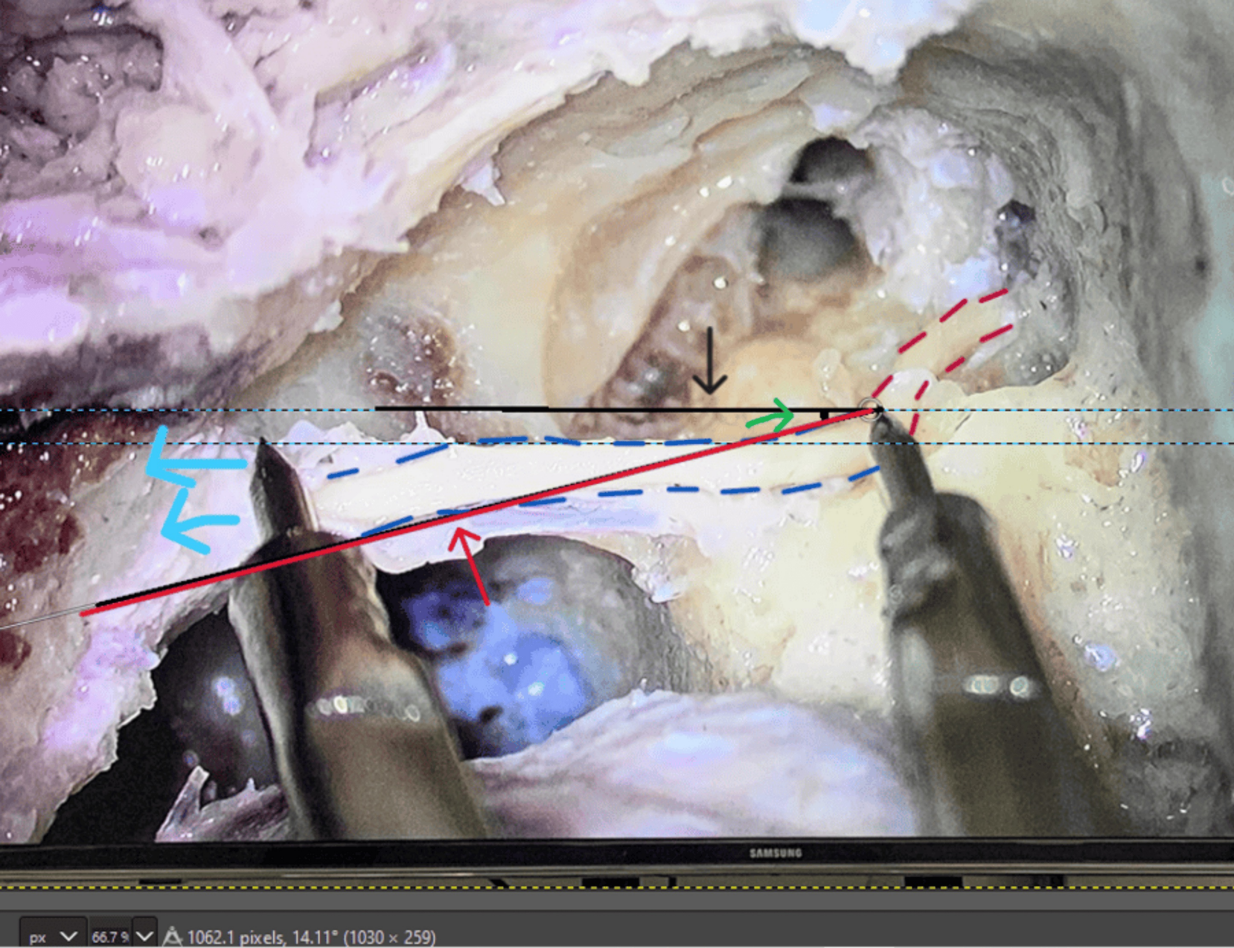
Measurement of the antero-posterior angle of the facial nerve along its exit toward the stylomastoid foramen: (a) Black arrow: horizontal line; (b) Red arrow: tangential line; (c) Green arrow: antero-posterior angle formed; (d) Blue arrows: direction of the stylomastoid foramen.

At the dome of the lateral semicircular canal, the tympanic segment of the facial nerve changes its directional course to become the mastoid segment of the facial nerve. At this point, using the GIMP2.10.36 software, a horizontal line was drawn. A tangential line was drawn at the point where the mastoid segment of the facial nerve enters the stylomastoid foramen. Both these lines were drawn in the sagittal plane. Using the same software, the angle formed by the facial nerve between the above-mentioned lines at the second genu was measured and calculated (Figure 4).

**Figure 5:**
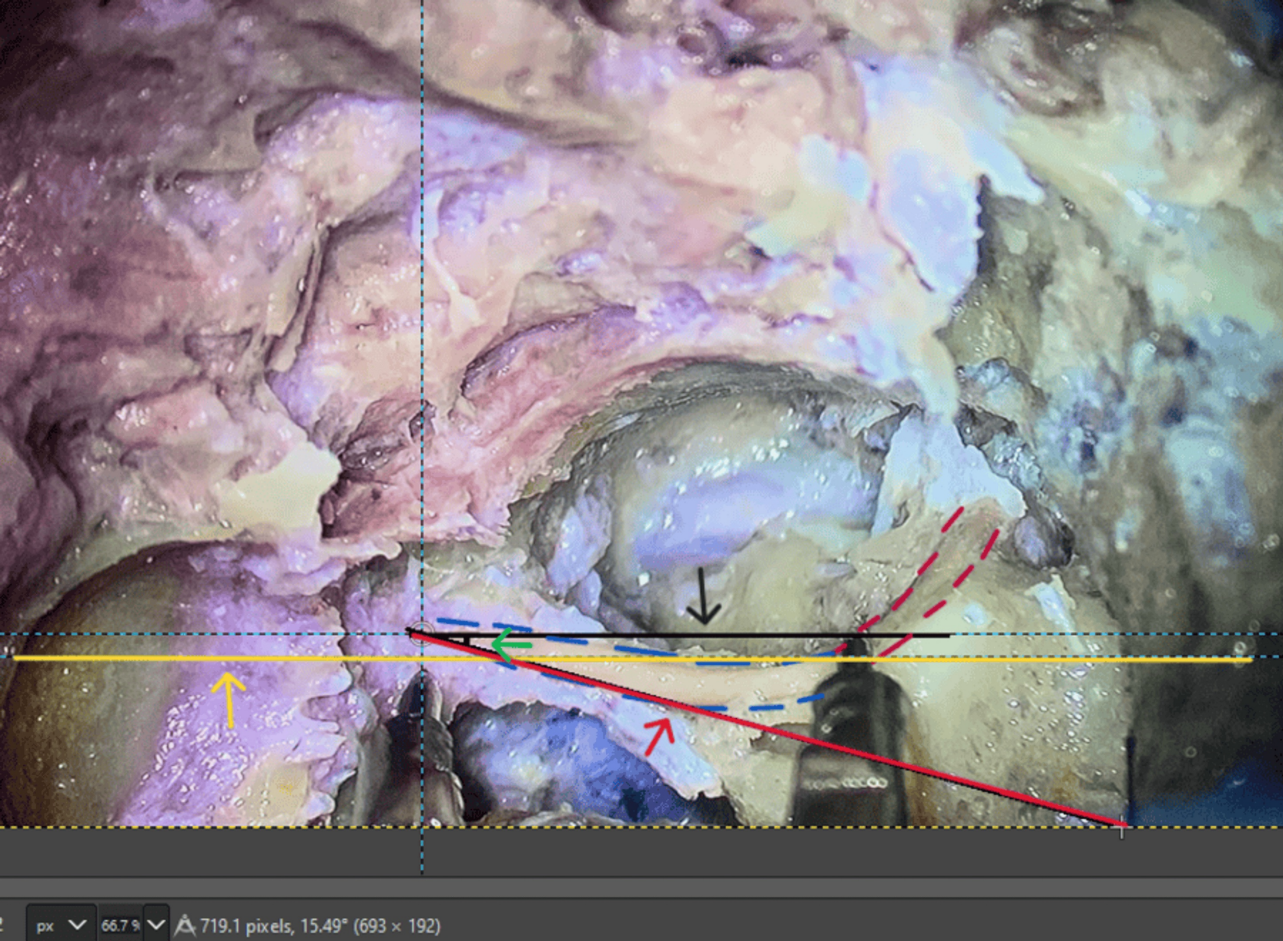
Measurement of medial to lateral angulation of the facial nerve as it exits the stylomastoid foramen: (a) Black arrow: first horizontal line parallel to the mastoid segment; (b) Yellow arrow: second horizontal line at the exit of the mastoid segment through the stylomastoid foramen; (c) Red arrow: third line tangential to the second line; (d) Green arrow: medial to lateral angle.

Similarly, using the same software, two horizontal lines were drawn: one parallel to the mastoid segment of the facial nerve at the level of the dome of the lateral semicircular canal and the second at the exit of the mastoid segment of the facial nerve at the stylomastoid foramen. A third line was drawn tangentially to the second parallel line. The angle formed at this point where the tangential line bisects the first horizontal line was measured and documented (Figure 5).

Similarly, all 20 specimens were dissected, and the anteroposterior and medial to lateral angulation was calculated, documented, and subjected to statistical analysis. p-values and paired t-tests were calculated and documented.

## Results

Out of the 20 wet temporal bone specimens dissected, 16 bones were found to be well pneumatised, and four bones were found to be sclerotic. The side of the temporal bone chosen was equal to avoid any bias: 10 were of the left temporal bone and 10 of the right temporal bone.

After the exposure of the facial nerve, its entire course was traced from the second genu to its exit at the stylomastoid foramen. It was observed that the tympanic segment of the facial nerve continued as the mastoid segment at the anterior wall of the mastoid portion of the temporal bone. The mean length of the mastoid segment was found to be 12.8 mm (±1.8 mm). The tympanomastoid suture line was medial to the mastoid section of the nerve [6]. The facial bony canal over the mastoid segment was found to be intact in all 20 specimens.

The antero-posterior and medial to lateral angulation of the facial nerve in both right and left temporal bones were calculated using the GIMP 2.10.36 software as given in Tables 1-2, respectively.

**Table 1:** Antero-posterior angulation (range) of the mastoid segment of the facial nerve as it exits the stylomastoid foramen in the right and left temporal bones.

Range of angulation (in degrees)	Right temporal bone	Range of angulation (in degrees)	Left temporal bone
10-12.9	2	10-12.9	0
13-15.9	5	13-15.9	7
16-18.9	3	16-18.9	3

**Table 2:** Medial to lateral angulation (range) of the facial nerve as it exits the stylomastoid foramen in the right and left temporal bones.

Range of angulation (in degrees)	Right temporal bone	Range of angulation (in degrees)	Left temporal bone
10-12.9	1	10-12.9	0
13-15.9	6	13-15.9	7
16-18.9	3	16-18.9	3

The mean antero-posterior angle measured at the level of the second genu was found to be 15.31˚, with 7 specimens having an angulation between 12-14˚, and 13 specimens having an angulation between 15-18˚. The mean medial to lateral angulation of the facial nerve as it exits the stylomastoid foramen was found to be 15.37˚, with 8 specimens having angulation between 11-14˚ and 12 specimens having angulation between 15-18˚. The p-value was calculated for both angles, and significance was interpreted.

The one-sample t-test was done to calculate the p-value and significance of antero-posterior and medial to lateral angulation with the standard cut-off value. The antero-posterior angle showed a p-value of 0.456, indicating that the sample did not significantly vary from the cut-off angle of deviation of the facial nerve. The SD was found to be 1.84. The p-value of the medial to lateral angle of deviation of the mastoid segment of the facial nerve was 0.275, which did not show any statistical significance on the t-test. The SD was found to be 1.47. This implies that the facial nerve, as it traverses the intratemporal region, can have a lot of variations in its course, and a particular value of angulation [15 degrees] cannot be considered as the standard cut-off value. This indicates that the nerve, especially the mastoid segment, is highly prone to iatrogenic facial nerve injury during surgery.

Interpreting the above results, it was found that the facial nerve courses more laterally and superficially by an angle of 15.37˚ towards its exit at the stylomastoid foramen. Hence, it is more prone to the risk of injury during mastoid surgeries.

## Discussion

Facial nerve is known to have an intricate course, which makes it challenging to preserve during surgeries like facial nerve decompression. In our study, we have emphasized the variable course of the mastoid segment of the facial nerve with respect to its angle of deviations throughout its intratemporal course until its exit towards the stylomastoid foramen where the nerve becomes more superficial. In literature, the anatomical course of the nerve along with its variations has been studied extensively, and various articles show its implications in practice [5]. There is still a paucity in literature regarding the course of this mastoid segment of the facial nerve and the angle that it travels by, and there have been no recent studies based on this. Hence, our study helps to bridge the gaps and provide insight into the highly variable course of the mastoid segment of the facial nerve.

Kharat RD et al. [6] conducted a study that highlighted the importance of the facial nerve during middle ear surgeries. They observed that the nerve had a very variable course, and the mastoid segment of the facial nerve was seen to become more superficial as it descends downwards towards the stylomastoid foramen, making it more prone to facial nerve injury causing facial paralysis.

According to Wiet RJ [7], operative facial paralysis accounted for the second most frequent cause of malpractice in modern otologic procedures, including tympanoplasty, canalplasty, hypotympanotomy, radical and modified mastoidectomy, and exostosis and osteoma excision. The facial nerve was visualized in a three-dimensional aspect where it made an angle as it deviated towards its exit at the stylomastoid foramen, keeping the second genu as its axis. This deviation was also documented by a study conducted by Jatale SP et al. [8] where the nerve shifts its course.

The importance of this angle is better understood by our study as a small change in angulation leads to a new path for the nerve, thereby putting the nerve at a variable risk of injury. This holds its importance as the mastoid segment of the facial nerve is most often incurred during otological surgeries and is prone to injury. Facial nerve injury has been reported in many major otological procedures such as cochlear implant, posterior tympanotomy, sinus tympani, and hypotympanic disease [9], and this insight will help avoid such events. The rate of facial nerve injury as described by Opus et al. was 6.2% in general ear surgeries and 18% in complex ear surgeries.

Nager GT and Proctor B [10] conducted a study that showed the facial canal may exhibit bony dehiscences, changes, and anomalies along its natural course as it passes through the temporal bone. There may be clinical and surgical implications for each of these characteristics.

Kullman GL et al. [11] conducted a study on the anatomy of the mastoid segment of the facial nerve in which they stated that the most accepted theory of the cause of Bell's palsy is due to dysregulation of the vascular system surrounding the facial nerve. The study showed that, in some cases, variations in the anatomy of the mastoid segment, crowding of the fallopian canal near the stylomastoid foramen by the chorda tympani nerve may be a factor for facial paralysis.

Kudo H and Nori S [12] reported that the mastoid segment of the facial nerve was concave medially and the beginning of this portion was situated laterally, compared with the end of the portion. Tracing the nerve becomes more difficult if its anatomy is not clearly and completely understood. It was found that there is a staggering 30% increase in facial nerve injury in younger surgeons compared to experienced ones. Hence, this angle acts as a guide for new budding surgeons to avoid iatrogenic neural injury.

In this study, we have identified the angle of deviation of the mastoid segment of the facial nerve, the antero-posterior and medial to lateral angulation as it courses through the intratemporal region towards its exit at the stylomastoid foramen, keeping the second genu as the axis. However, there are certain limitations associated with the study like a small sample size, lack of a standard technique for the measurement of the angle of deviation of the facial nerve leading to bias in the measurement of the angulation, and paucity of literature supporting the results.

## Conclusions

The intratemporal course of the facial nerve exhibits a lot of variations, especially in the mastoid segment. Therefore, intricate anatomical knowledge, along with meticulous dissection of the nerve, becomes extremely important in avoiding complications during mastoid surgeries. Our study showed that the mastoid segment of the facial nerve has a highly variable course across different age groups, owing to its anatomical development. The deviation in angulation of the mastoid segment can be easily measured by surgeons, which helps prevent the risk of injury to the facial nerve during middle ear surgeries. It also emphasized the importance of having sound anatomical knowledge about the various anatomical relations of the nerve, which helps prevent facial nerve injury.

This angle of deviation of the mastoid segment of the facial nerve from the second genu to the stylomastoid foramen will act as a guide for surgeons and caution them against any inadvertent iatrogenic facial nerve injury.
